# The Roles of General and Domain-Specific Perceived Stress in Healthy Aging

**DOI:** 10.1093/geroni/igab046.037

**Published:** 2021-12-17

**Authors:** Jing Luo, Bo Zhang, Emily Willroth, Daniel Mroczek, Brent Roberts

**Affiliations:** 1 Northwestern University, Chicago, Illinois, United States; 2 Texas A&M University, College Station, Texas, United States; 3 University of Illinois-Urbana-Champaign, Champaign, Illinois, United States

## Abstract

Theoretical and empirical evidence suggests the existence of a general perceived stress factor overarching different life domains. The present study investigated the general perceived stress relative to domain-specific perceived stress as predictors of 26 diverse health outcomes, including mental and physical health, health behaviors, cognitive functioning, and physiological health indicators. A bifactor exploratory structural equational modelling approach was conducted in two samples from the Health and Retirement Study. Across the two samples, perceived stress was well-represented by a bifactor structure where there was a robust general perceived stress factor representing a general propensity towards stress perception. Meanwhile, after controlling for the general factor, specific factors representing perceived stress in different life domains were still clearly present. The general perceived stress factor was the most robust predictor of the majority of health outcomes. Age, sex, personality traits, and stressor exposure were found as possible diathesis underlying the general perceived stress factor.

